# Psychiatry and psychotherapy: the science of sitting with uncertainty

**DOI:** 10.1007/s11845-024-03687-5

**Published:** 2024-04-23

**Authors:** Aoibheann McLoughlin

**Affiliations:** University College Dublin, Belfield, Dublin 4, Ireland

**Keywords:** Psychiatry, Psychotherapy, Uncertainty, Science

## Abstract

**Objective:**

This perspective piece aims to delve into the challenges and possibilities arising from uncertainty in psychiatry and psychotherapy.

**Method:**

This is a perspective piece.

**Results:**

Historical considerations, polarised conceptual frameworks, interacting systems, limited randomised controlled research, and varying practice approaches, coalesce to form an exoskeleton of ambiguity and uncertainty in psychiatry and psychotherapy that seems almost impenetrable. Yet it is these very things that allow psychiatry to challenge accepted norms, avoid complacency, and shed its skin.

**Conclusion:**

Ambiguity and uncertainty in the setting of partial knowledge presents psychiatry with a challenge, yes; but it also enables psychiatry to be innovative in its approach, and to address the complexities of the human condition in a way that is unique and unparalleled within the field of medicine.

As a trainee, I have struggled at times to fully embrace the uncertainty and ambiguity that exists within the structure and practice of psychiatry. Since its inception over the past four centuries, psychiatry has been in a state of ecdysis, shedding its old skin and forming anew. Yet an exoskeleton of uncertainty pervades. From its “moral treatment” origins in the eighteenth century to the biological systemisation of the nineteenth century, through to the advent of psychodynamic theory in the 1890s, and the rise of psychopharmacology in the mid-twentieth century, psychiatry has navigated a varied path through its history. This course extended in the latter part of the twentieth century to include a social and community-based focus, and has more recently incorporated the emergence of neuroscience and genetics. Looking at its history then, the tapestry of psychiatry is woven of composite and ideologically varied strands that encompass distinct philosophical, cultural, biological, and psychosocial movements which have contributed towards contemporary practice.

Throughout this history, the pull between the logical positivism of science [1] versus existential thinking in psychiatry has led to psychiatrists practicing within a milieu of opacity. Unlike the use of biological or physiological markers to diagnose specific conditions across medicine, psychiatry relies on subjective interpretation of signs and symptoms, along with observation of course of illness to provide a basis for intervention [2]. Psychiatry is viewed to be hindered by a lack of valid predictive biomarkers that could aid objective diagnosis and support targeted individualised treatment [3]. Interventions in psychiatry are not uniform across psychotherapeutic or pharmacotherapy realms. Yes, there are guidelines and algorithms for diagnosis and treatment, yet what may be indicated by an evidence base may not fit the specific needs of the client. In pharmacotherapy, drug tolerance, side effects, and drug interactions affect clinical course and outcome. In psychotherapy, the multivariate concepts, techniques, and applications of myriad practice approaches ranging from the psychodynamic to the behavioural can be overwhelming to patients and professionals alike [4].

Like interventions, outcomes in psychiatry are far from constant, with specific challenges inherent in the measurement of generalisable results. While evidence-based practice tends to centre on the amelioration of symptoms, in psychotherapy, measuring the subjective sense of enhanced support, empowerment, and self-discovery can be problematic in the quantitative domain of research. This has led to a preponderance of a cognitive-behavioural therapy evidence base due to the ready availability of measurable outcomes within this form of therapy. However, a person’s progress in psychotherapy is often subjectively determined and will reflect the heterogeneity of the client population and the dynamic internal and external factors that contribute towards wellness and recovery. Environmental and cultural factors certainly play a role, as do genetics, substance use, interpersonal style, financial concerns, personality constructs, and help-seeking patterns [4]. These variables add an extra layer of complexity to the treatment and recovery process and heighten the ambiguity around outcomes for patients with mental illness.

Psychiatry’s bridging role between the humanities and the biological sciences [5] offers a unique approach to assessment and treatment, yet its validity has become attenuated in response to the view that it is lacking an empirical robustness afforded to pure biological enquiry. Psychiatry has thus come to occupy an increasingly tenuous position within medicine, with an external societal ambivalence mirroring that of its internal ambiguity. Yet mental disorders arise in the context of overlapping and interacting systems that encompass neurobiological, psychological, experiential, environmental, risk, and resilience spheres that evolve over time and are embedded within each other [6]. Embracing the systemic complexity of psychiatry requires an interpretation that extends beyond reductionist conceptualisations of mental health and explores the variability that emerges within the setting of these multifactorial elements [7]. Such complexity poses a challenge for predictive models, which are highly context-specific in psychiatry, and thus will have implications in terms of generalisability [8].

Reliant, in part, on phenomenological observation, intuitive interpretation, and the application of diverse approaches to assessing signs and treating symptoms, the psychotherapeutic domain of psychiatry has remained an elusive entity to pin down scientifically. Psychotherapeutic interventions thus became devalued due to a lack of documented randomised controlled proof of efficacy. The characteristics and conditions of randomised controlled treatment outcome research in psychotherapy in comparison to real-world clinical practice to date have been viewed to be flawed, due to a lack of emphasis on individual factors affecting patient health, dearth of clinical relevance, under-representation of minorities and those with complex heath needs, and avoidance of potential confounders [9]. As such, critiques of psychiatry question the capacity of psychotherapeutic interventions to bring about tangible and measurable outcomes for patients. Perhaps, this has led to the fervent embrace of biological psychiatry in the form of genetic enquiry and neuroscience as it seeks validation from the scientific community through the search for scientific fact, precision, and certainty.

Evidence-based medicine has progressed in a bid to ensure best possible outcomes for patients and to eliminate clinical uncertainty via standardisation of practice guidelines. Yet no guidelines exist that will have 100% sensitivity and specificity. If purely scientific methods were applied to psychotherapy, then certain elements of therapy would be associated with definite outcomes. Psychiatry, by virtue of its analysis of the human condition however, can never achieve absolute certainty. It demands a nuanced exploration of the underlying surface of the mind in order to allow the implicit to become truthfully explicit and enable the patient to work towards recovery and self-actualisation. While it may be easier to work in the calming comfort of certainty, where results meet tested expectations, the human condition demands a more complex approach devoid of hubris. The quest for certainty would dilute the role of professional judgement, intuition, compassion, and creativity in arriving at a clinical decision.

Scientific enquiry does not always equate to incontrovertible fact. Take Heisenberg’s Uncertainty Principle, for example. Werner Heisenberg (1901–1976) discovered that in quantum mechanics, the position and momentum of an object could not be measured concurrently with absolute precision. As such, the more precisely the position of a particle was ascertained, the less precisely its velocity could be determined. The same applied contrawise. This principle is seen as one of the accepted uncertainties in modern-day science and an example that, even in science, there are no absolutes. Taking this into account, we could view psychiatry as a medical proponent of the Heisenberg Principle. Within the therapeutic encounter (due to internal and external processes that unfold), the more we think we know about a patient (position), the less certain we may feel in terms of the course of action needed in order to facilitate progress (momentum/velocity). Nothing is certain. Not in psychiatry and not in science.

Historical considerations, cultural factors, nosology structures, polarised conceptual frameworks, limited randomised controlled research, interacting systems, and varying practice approaches coalesce to form an exoskeleton of ambiguity and uncertainty in psychiatry that seems almost impenetrable. Yet it is these very things that allow psychiatry to challenge accepted norms, avoid complacency, and shed its skin. The task of knowledge attainment, accurate formulation, and effective treatment given the unique and multivariate complexities of the human condition is not Sisyphean. The challenge is not something to be tolerated, rather something to be embraced. The quest for enhanced knowledge in the face of uncertainty is what drives a need for better understanding of internal and external factors affecting patients, ultimately leading to improvement in diagnostic and clinical approaches.

Ambiguity and uncertainty in the setting of partial knowledge presents psychiatry with the chance to be innovative in its approach and to address the complexities of the individual human condition in a way that is unique and unparalleled within the field of medicine. There is wisdom in accepting the role of the naïve explorer who does not have all the answers, but will endeavour to be an inquisitive conduit towards self-discovery. The challenge is to inhabit the uncertainty, allow the ambiguity to energise discovery, and channel it into enhancing communication with patients in promoting a path towards recovery.
